# HLA-DM Mediates Epitope Selection by a “Compare-Exchange” Mechanism when a Potential Peptide Pool Is Available

**DOI:** 10.1371/journal.pone.0003722

**Published:** 2008-11-13

**Authors:** Andrea Ferrante, Matthew W. Anderson, Candice S. Klug, Jack Gorski

**Affiliations:** 1 Blood Research Institute, Blood Center of Wisconsin, Milwaukee, Wisconsin, United States of America; 2 Department of Biophysics, Medical College of Wisconsin, Milwaukee, Wisconsin, United States of America; University of Cambridge, United Kingdom

## Abstract

**Background:**

HLA-DM (DM) mediates exchange of peptides bound to MHC class II (MHCII) during the epitope selection process. Although DM has been shown to have two activities, peptide release and MHC class II refolding, a clear characterization of the mechanism by which DM facilitates peptide exchange has remained elusive.

**Methodology/Principal Findings:**

We have previously demonstrated that peptide binding to and dissociation from MHCII in the absence of DM are cooperative processes, likely related to conformational changes in the peptide-MHCII complex. Here we show that DM promotes peptide release by a non-cooperative process, whereas it enhances cooperative folding of the exchange peptide. Through electron paramagnetic resonance (EPR) and fluorescence polarization (FP) we show that DM releases prebound peptide very poorly in the absence of a candidate peptide for the exchange process. The affinity and concentration of the candidate peptide are also important for the release of the prebound peptide. Increased fluorescence energy transfer between the prebound and exchange peptides in the presence of DM is evidence for a tetramolecular complex which resolves in favor of the peptide that has superior folding properties.

**Conclusion/Significance:**

This study shows that both the peptide releasing activity on loaded MHCII and the facilitating of MHCII binding by a candidate exchange peptide are integral to DM mediated epitope selection. The exchange process is initiated only in the presence of candidate peptides, avoiding possible release of a prebound peptide and loss of a potential epitope. In a tetramolecular transitional complex, the candidate peptides are checked for their ability to replace the pre-bound peptide with a geometry that allows the rebinding of the original peptide. Thus, DM promotes a “compare-exchange” sorting algorithm on an available peptide pool. Such a “third party”-mediated mechanism may be generally applicable for diverse ligand recognition in other biological systems.

## Introduction

Antigen presentation to CD4+ T lymphocytes by major histocompatibility complex class II (MHCII) molecules is determined by a series of complex cellular and molecular events occurring within antigen presenting cells. Proteins derived from the secretory or endocytic pathway are proteolytically cleaved into peptide fragments and loaded into MHCII within specialized endosomal vesicles termed MHC class II compartments (MIIC). The binding of a peptide to the MHCII involves interactions between peptide side chains and pockets lining the MHCII peptide binding groove as well as a conserved, extensive hydrogen bond (H-bond) network between the peptide backbone and the MHCII [1].

MHCII molecules are transported from the endoplasmic reticulum to the MIIC as nonameric complexes with the chaperone protein invariant chain (Ii). Ii stabilizes the nascent MHCII and prevents the binding of other endoplasmic reticulum-resident peptides. Upon arrival in the MIIC, the Ii molecule is cleaved through the action of proteases leaving a peptide fragment termed CLIP in the MHCII peptide-binding groove [2]. CLIP is then released by the action of a class-II like molecule called HLA-DM (DM) to allow antigenic peptide to bind MHCII [3]–[6]. DM's exchange role is not limited to CLIP as it can catalyze the exchange of antigenic peptide to select for a kinetically stable peptide/MHCII repertoire [7]–[10]. DM has also been shown to stabilize unbound MHCII from the irreversible loss of the peptide binding site or rescue misfolded MHCII from denaturation and aggregation [11], [12].

DM mediated peptide exchange is usually examined in terms of one of its functions, enhancing the release of the prebound peptide. In this regard, several studies have identified possible interaction sites between MHCII and DM during peptide release [13], [14]. Other data has shown the importance of H-bonds in the N-terminal region of the peptide binding groove for peptide/MHCII stability [15] and the possible role of DM in the destabilization of this region [16]. The importance of a conserved H-bond at residue 81 in the MHCII β chain in peptide/MHCII complex stability has also been shown in the absence [17] and presence of DM [18].

Studies of peptide exchange in the absence of DM have proven to be surprisingly complex for a non-covalent ligand/receptor interaction. One complicating factor is the important observation that the relatively slow level of spontaneous peptide release can be enhanced by the presence of a second peptide, a phenomenon referred to as “pushing off” [19]. Indeed, the existence of a two peptide–MHCII intermediate in this process was suggested by fluorescence energy transfer (FRET). However, the effect of a second peptide on DM-mediated peptide exchange reaction has not been investigated. The ability of DM to stabilize an open conformation of MHCII or recover partially denatured or aggregated MHCII may also play a role in the mechanism of peptide exchange. Models invoking this function propose that DM preferentially interacts with empty MHCII and catalyzes a conformational change which facilitates the binding of available peptides [11], [12], [20].

One way to approach the problem of complexity in peptide/protein interactions is through the analysis of cooperative effects during binding and dissociation. We have previously shown that in the absence of DM, the binding of peptide to HLA-DR1 can be described using a cooperative model in which the contribution of a specific residue to peptide/MHCII affinity is dependent upon peptide/MHCII interactions throughout the binding site [21], [22]. We consider cooperativity as reflecting the folding process through which the peptide and the binding groove may achieve a stable conformation, consistently with the interpretation given in other systems [23]. Cooperative effects throughout the peptide binding site would also provide an explanation for the fact that both types of binding energy available to the peptide/MHCII complex (hydrophobic interactions and hydrogen bonding) can influence DM stability [16], [24]. Under a cooperative model of peptide/MHCII interactions, DM would discriminate among peptide sequences based on the total binding energy resulting from distributed interactions across the peptide-binding groove. Indeed, peptide substitution of solvent-exposed side chains, as well as modification of the P1 pocket interaction affect complex stability in the presence of DM [25].

Here we investigate whether DM action on the complex affects cooperativity and how this may be related to the mechanism underlying the peptide exchange reaction. First we show that cooperative effects are only measured at the level of the exchange peptide. An important role for the exchange peptide is further supported by the lack of efficient peptide exchange in the absence of a high affinity exchange peptide at sufficient concentration. Increased FRET signal between the bound and exchanging peptide in the presence of DM provides support for the requirement of a tetramolecular intermediate in the mechanism of DM-mediated exchange. Our results suggest that DM mediates epitope selection by a “compare-exchange” sorting process in which bound peptide release occurs only in the presence of exchange peptides, and these are tested by MHCII on the basis of their folding properties in the context of a geometry that allows for rebinding of the original peptide.

## Results

### Absence of cooperativity in DM-mediated peptide release of the prebound peptide

In our previous work, we have shown that the transition between the empty and bound conformer of peptide/MHCII complexes in the absence of DM is cooperative; in that the total binding energy of the complex is dependent on distributed interactions across the peptide-binding groove [21], [22]. Moreover, previous kinetic analyses of DM function have suggested that DM acts as a conformational catalyst to promote the conversion between the empty and bound conformation of the peptide/MHCII complex [16]. Therefore we investigated how the presence of DM may impact cooperativity, i.e. the folding/unfolding of the complex.

A general strategy for the direct analysis of cooperativity is the mutant cycle approach devised by Fersht and colleagues [23], [26]. The experimental approach involves introducing a defined number of substitutions into the amino acid sequence of a protein. Next, the individual contribution of each substitution to the energetics of protein folding or catalytic activity is measured. Cooperativity between protein subunits is evidenced by a disproportionate effect in the presence of multiple substitutions than predicted by an analysis of each individual mutation.

To measure cooperative effects in DM-mediated peptide exchange, we utilized a series of hemagglutinin (HA) peptides substituted at the P2, 3, 7, and 10 positions, which are postulated to mediate their negative effects on affinity by interfering with the hydrogen bonding network [25]. In brief, molecular modeling studies suggest that the P2 Val to Ser substitution would affect H-bonds between β-82 Asn and the P2 amide and carbonyl groups. The P3 mutation, Lys to Asp, is postulated to destabilize the H-bonds between α9 Gln and α62 Asn with the carbonyl of P4. The substitution at P10, Val to Gly, is postulated to disrupt the H-bond between α76 and the P10 carbonyl. In addition to direct interference of hydrogen bonding, these substitutions may act indirectly to destabilize hydrogen bond interactions by increasing solvent accessibility. The P7 Leu to Pro substitution likely mediates its affect on affinity through steric interactions at the shallow hydrophobic pocket at the P7 position [27].

**Figure 1:**
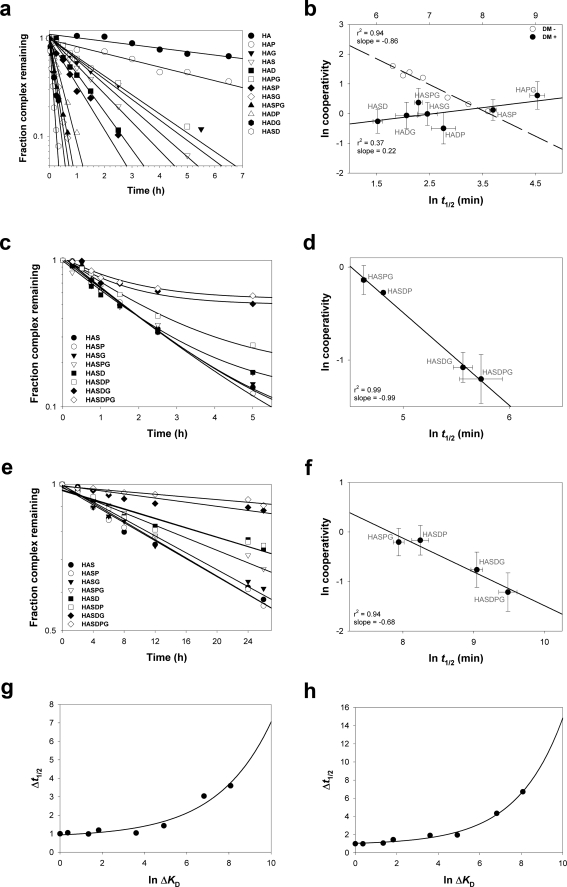
Cooperative effect on peptide dissociation from HLA-DR1 in presence of DM is evidenced at the level of the exchange peptide. (a) Dissociation rate of peptides from HLA-DR1 in the presence of DM was measured as described in Results. Data is plotted as the fraction of DR1/labeled peptide complex remaining relative to *t* = 0. Reactions were performed in triplicate, and data points represent one of two independent experiments. Lines fit the data to a single exponential decay function. (b) Natural log (ln) plot of cooperativity (expected/observed *t*_1/2_) vs. DM-mediated (solid line) and intrinsic (dashed line) dissociation rate for each DR1/peptide complex tested. To facilitate the comparison, data points were plotted on different scales for *t*_1/2_ values. Top x-axis scale refers to intrinsic dissociation rate. Bottom x-axis scale refers to DM-mediated off rate. Since we defined cooperativity *C* as the ratio of the expected to observed values for Δ*t*_1/2_, and *t*_1/2_ is directly proportional to stability, the cooperative effect is positive if 0≤*C*<1, while if C>1 the cooperative effect is negative. In the ln plot, positive cooperativity in stability is indicated on the y-axis by values <0 and negative cooperativity by values >0. Horizontal error bars represent the SD of the *t*_1/2_ measurement. Vertical error bars represent the error of cooperativity as calculated through SE propagation. Lines indicate the fit of the data to a linear regression. (c) DM-mediated dissociation of the HAS peptide from DR1. The nature of the competing peptide present in excess during the reaction is identified in the legend. Data points represent the mean of two independent experiments, and lines represent the fit of the data to a two or three parameter single exponential decay function. (d) Natural log (ln) plot of cooperativity (expected/observed t_1/2_) vs. dissociation rate of DR1/HAS complex for each multiple substituted exchange peptide tested. Error bars are as in panel B. The line indicates the fit of the data to a linear regression. (e) Intrinsic dissociation of the HAS peptide from DR1. The nature of the competing peptide present in excess during the reaction is identified in the legend. Data points represent the mean of two independent experiments, and lines represent the fit of the data to a two or three parameter single exponential decay function. (f) Natural log (ln) plot of cooperativity (expected/observed t_1/2_) vs. dissociation rate of DR1/HAS complex for each multiple substituted exchange peptide tested. Error bars are as above. The line indicates the fit of the data to a linear regression. (g) The ratio of *t*_1/2_ for the DR1/HAS complex measured in the presence of different exchange peptides and DM as compared to the *t*_1/2_ measured in the presence of HAS is plotted as function of the natural log of exchange peptide *K*_D_. The line indicate the fit of the data to an exponential function (r^2^ = 0.98). (h) The ratio of *t*_1/2_ for the DR1/HAS complex measured in the presence of different exchange peptides and in absence of DM as compared to the *t*_1/2_ measured in the presence of HAS is plotted as function of the natural log of exchange peptide *K*_D_. The line indicate the fit of the data to an exponential function (r^2^ = 0.97).

We first asked whether cooperative effects in the presence of DM would be measurable at the level of the peptide prebound in the HLA-DR1 (DR1) groove. Dissociation rate data for these peptides in the presence of DM are shown in **Figure 1a** and the *t*_1/2_ values are reported in **Table 1A**. Cooperative effects were calculated by determining the ratio of expected *t*_1/2_ to observed *t*_1/2_ for each substituted peptide relative to the off-rate of the DR1/HA complex in the presence of DM. When cooperativity was plotted against the observed *t*_1/2_ of the various DR1/peptide complexes (**Figure 1b**, solid line), we found a very poor correlation (r^2^ = 0.37), with a slightly positive slope. Interestingly, this was in contrast to the negative slope of cooperativity vs. off-rate in the absence of DM (**Figure 1b**, dashed line from ref. 21). Therefore, peptide release in the presence of DM is different from the typical unfolding process observed in the absence of DM.

**Table 1:** Dissociation rate of substituted peptide/DR1 complexes.

Complex	Exchange Peptide	Abbreviation (text and figures)	t_1/2_ (min)
**A**			
DR1/HA 306-319	HA (306-319)	HA	447.74±75.4
DR1/P2 V→S		HAS	60.32±11.18
DR1/P7 L→P		HAP	267.8±46.2
DR1/P2,7 VL→SP		HASP	40.36±2.84
DR1/P10 A→G		HAG	69±14.4
DR1/P7,10 LA→PG		HAPG	92.9±13.1
DR1/P3 K→D		HAD	43.78±6.12
DR1/P2,10 VA→SG		HASG	11.72±2.18
DR1/P2,7,10 VLA→SPG		HASPG	9.9±0.7
DR1/P3,7 KL→DP		HADP	15.87±3.63
DR1/P2,3 VK→SD		HASD	4.6±0.4
DR1/P3,10 KA→DG		HADG	7.92±1.64
DR1/P3,7,10 KLA→DPG		HADPG	*
DR1/P2,3,7 VKL→SDP		HASDP	*
DR1/P2,3,10 VKA→SDG		HASDG	*
DR1/P2,3,7,10 VKLA→SDPG		HASDPG	*
**B**			
DR1/P2 V→S	DR1/P2 V→S	HAS	85.8±10.1
	DR1/P2,7 VL→SP	HASP	90.6±12.4
	DR1/P2,10 VA→SG	HASG	85.1±9.1
	DR1/P2,7,10 VLA→SPG	HASPG	102.4±2.5
	DR1/P2,3 VK→SD	HASD	89.6±5.2
	DR1/P2,3,7 VKL→SDP	HASDP	123.1±1
	DR1/P2,3,10 VKA→SDG	HASDG	260.7±26.3
	DR1/P2,3,7,10 VKLA→SDPG	HASDPG	309.0±49
**C**			
DR1/P2 V→S	DR1/P2 V→S	HAS	1950±183
	DR1/P2,7 VL→SP	HASP	1930±164
	DR1/P2,10 VA→SG	HASG	2080±233
	DR1/P2,7,10 VLA→SPG	HASPG	2810±216
	DR1/P2,3 VK→SD	HASD	3720±279
	DR1/P2,3,7 VKL→SDP	HASDP	3810±453
	DR1/P2,3,10 VKA→SDG	HASDG	8470±700
	DR1/P2,3,7,10 VKLA→SDPG	HASDPG	13100±1684

* Not measured.

### Cooperativity is observed in the exchange peptide during DM-mediated exchange

An integral aspect of measuring *K*_D_, intrinsic off rates and DM-mediated release is the presence of an exchange peptide, also called “competitor”. This is usually an unlabeled peptide nominally added in excess to the reaction to prevent the rebinding of freshly dissociated labeled peptide. However, the presence of the exchange peptide is a parameter whose effect is incompletely understood. To address the role of the exchange peptide in DM activity, we selected a peptide which had an appreciable off-rate in the presence of DM. In our previous experiments, a HA peptide containing a Ser for Val mutation at P2 (P2 V→S) (HAS) was found to have a small effect on affinity as compared to wild-type HA, but significantly increased dissociation rate in the presence of DM [25]. DR1/HAS complexes were generated and off-rates were measured in presence of DM and in the presence of 100-fold excess of different exchange peptides (**Figure 1c** and **Table 1B**). To control for the effect of the P2 (V→S) mutation, each exchange peptide tested also contained the P2 mutation.

The half-life values were used to calculate cooperativity relative to the exchange peptide. We found cooperativity with a high correlation (r^2^ = 0.99) and with a slope of −0.99 (**Figure 1d**). In addition, the relationship between cooperativity and *t*_1/2_ was similar to that obtained in the absence of DM when cooperativity was defined relative to the bound peptide (**Figure 1b**, open circles). Since all the substitutions introduced in the HA sequence are postulated to destabilize binding, our assays measure a negative cooperative effect on complex stability. These results suggested that during peptide exchange in presence of DM, the exchange peptide may replace the prebound peptide based on its ability to form a stable conformer of the peptide/MHCII complex.

The observation that cooperativity was detected in the exchange peptide during DM-mediated exchange raised the question whether cooperativity could be observed in the absence of DM. To address this issue we measured DR1/HAS complex stability in the same conditions but without DM. Dissociation rate data are shown in **Figure 1e** and the *t*_1/2_ values are reported in **Table 1C**. Interestingly, when we calculated the values of cooperativity for the exchange peptide in the absence of DM, we found a similar negative slope as measured in the presence of DM (**Figure 1f**) but to a lesser extent (slope of −0.68 vs. −0.99).

### The affinity of the exchange peptide for the DR1 binding groove affects DM mediated peptide release

The cooperativity data indicated a mechanistic relationship between the non-cooperative release of the prebound peptide and the cooperative binding of the exchange peptide. Interestingly, the off-rate experiments in **Figure 1c and 1e** also show that the *t*_1/2_ of the DR1/HAS complex varied considerably based on the affinity of the exchange peptide for DR1. When a HA-derived peptide with a single or a double substitution was present in excess, the dissociation rates of the prebound peptide were similar, with a *t*_1/2_ of approximately 85–90 min (**table 1B**). However when the affinity of the exchange peptide was decreased through multiple substitutions, we found that the stability of the DR1/HAS complex was significantly increased (309 min for HASDPG, **table 1B**). A similar trend could be observed when complex stability was measured in absence of DM. When the normalized *t*_1/2_ values of DR1/HAS complex were plotted against the normalized *K*_D_ of the various exchange peptides [21], we found a positive exponential correlation either in the presence (**Figure 1g**) or in the absence of DM (**Figure 1h**). These data indicate that the exchange peptide promotes prebound peptide release at a rate which is a function of its affinity for DR, and DM affects the exchange reaction through enhancement of this effect.

### DM mediated peptide exchange is dependent on an exchanging peptide

In our previous experiments, the effect of the exchange peptide was measured indirectly, by monitoring the release of labeled prebound peptide. To more directly study the relationship between the exchange peptide and release of prebound peptide, we next asked whether DM would release prebound peptide in the absence of an exchange peptide. This required an experimental approach wherein the amount of free peptide would be directly measured over longer time scales.

To this end, we utilized two different approaches to examine the dependence of peptide release on the presence of exchange peptide; electron paramagnetic resonance (EPR) and fluorescence polarization (FP). The rationale for both methodologies was to start with a prebound peptide/MHCII complex and observe the accumulation of free peptide over time in the absence of an exchange peptide, DM, or both. The missing component(s) was then added to the reaction, and the exchange rate was monitored.

**Figure 2:**
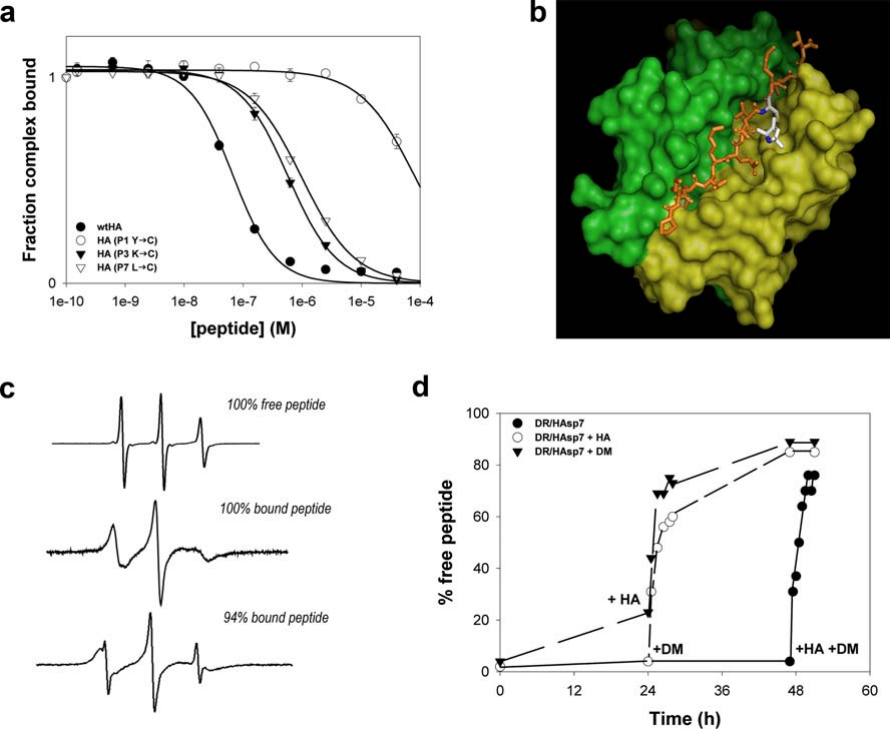
Free peptide is a co-factor in DM-mediated peptide release. (a) Competitive binding analysis of P1, P3 and P7 MTSL-Cys substituted HA peptide variants to DR1. Data represent the mean and SD of three independent experiments. Lines indicate the fit of the data to a logistic equation. *K*_D_ values for the peptides as listed in the legend are respectively 83.3 nM, 28.8 μM, 237.9 nM and 404.7 nM. (b) Top view of the HA peptide P7 Leu→Cys substituted, labeled at this position with MTSL (HAsp7) and complexed with HLA-DR. The peptide is in orange, the α-chain is in green and the β-chain in yellow. The most energetically favored orientation of the probe (white) is shown. The model was generated using PyMol [45]. Coordinates taken from ref. 41. (c) Spectra of HAsp7 peptide as recorded when free in solution (top), completely bound to HLA-DR (center) or as a composite of the two states (bottom). The broadening of the spectral lines and a slight shift in their positions in the spectrum appear evident. (d) Dissociation of HAsp7 peptide from DR1 was measured as described in Results. Data is plotted as the % of free (unbound) peptide detected at each time point as quantitated by spectral subtraction methods. Data points represent one of three independent experiments. Data points referring to the C sample at *t* = 0 and *t* = 24 are hidden below other data points.

For the EPR studies we utilized a site directed spin labeling (SDSL) approach coupled with EPR spectroscopy [28], to measure the dissociation from HLA-DR1 of a peptide derived from the HA peptide. The HA peptide was substituted with cysteine at P1, P3 or P7 and labeled with MTSL, which introduces a stable free electron-containing nitroxide moiety at the cysteine residue (see methods). The modification at P3 and P7 resulted in a 3- and 5-fold decrease in peptide binding to DR1 respectively, as measured through a competition binding assay (**Figure 2a**), but still allowed for stable complex formation. We selected the P7 substituted peptide for further experiments, as the P7 L→C substitution resides in a shallow hydrophobic pocket [27] which might be expected to restrict rotation of the spin-label probe (HAsp7) (**Figure 2b**). In EPR analysis, the labeled substrate undergoes a resonant absorption of microwave radiation in the presence of a static magnetic field. The resultant spectra are dependent on the motion of the spin-label side-chain. Rapid tumbling of the spin label in solution gives rise to three narrow lines (**Figure 2c** top). As the rotational freedom of the probe becomes restricted (*i.e.* upon MHCII binding), the EPR spectrum shows broadening of the spectral lines and a shift in their relative positions within the spectrum (**Figure 2c** center). EPR spectra are composites of all motions present within a sample (e.g. **Figure 2c** bottom), and not an average, thus peptide dissociation can be monitored and quantified in real time without a separation step.

Spectra were acquired in the following reactions: DR1/HAsp7 in the presence of 3-fold molar excess DM; DR1/HAsp7 complexes in the presence of 100-fold excess unlabeled HA; and DR1/HAsp7 complexes alone. As shown in **Figure 2d**, the amount of free peptide present at *t* = 0 hr averages 3% for all reactions tested, confirming that nearly all of the HAsp7 peptide is bound to DR1. After 24 hr, there was negligible release in the presence of excess HA. Approximately 20% free peptide was observed in the presence of DM. At this point, soluble DM was added to the reaction with excess HA, and 100-fold excess unlabeled HA peptide was added to the reaction with DM, and incubation continued. Four hours after the addition of either DM or peptide to the respective reactions, we observed an equivalent increase in free HAsp7 peptide (60% over the 24 hr timepoint). Furthermore, at 48 hr, when both an exchange peptide and DM were added simultaneously to the incubation with DR1/HAsp7 complex alone (**Figure 2d**, closed circles), we observed a similar magnitude of peptide release (76%). This confirmed that the DR1/HAsp7 complex was stable and maintained the ability to undergo peptide exchange after long incubation periods. The rate of DM-mediated spin-labeled peptide release during the 4 hour incubation in presence of excess unlabeled exchange peptide shows a three-fold increase over the 24 hr incubation without exchange peptide (60% vs. 20%) loss, suggesting that, under the conditions tested, the exchange peptide plays a role as a co-factor in the DM-catalyzed off rate.

### FP analysis of the role of free peptide in DM-mediated peptide dissociation

The acquisition of an EPR spectrum requires multiple scans (up to 25). As such, each time point for a particular reaction represents the average of several measurements. To more precisely measure peptide exchange, we performed a similar experiment using fluorescence polarization (FP). FP methodology takes advantage of the fact that the light emitted by a fluorophore upon excitation with plane polarized light is polarized as well. The angle between the planes of exciting and emitted light is dependent on the tumbling of the fluorophore. Therefore, if viscosity and temperature are held constant, polarization is directly related to the molecular volume. If a fluorescent probe binds to a molecule with a higher molecular weight, this average angle will decrease due to the slower molecular rotation of the bound probe. Thus, the ratio between bound and free peptide can be measured directly in solution without a separation step [29].

**Figure 3:**
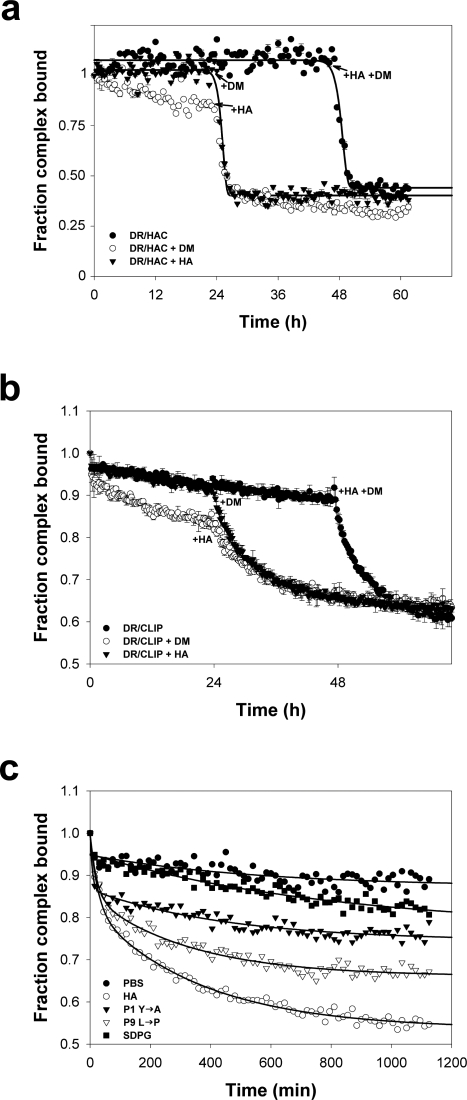
FP analysis of the role of free peptide in DM-mediated peptide dissociation. (a) Real-time analysis of DR1/HAC stability as described in Results. (b) Real-time FP analysis of DR1/CLIP stability as described in Results. For (a) and (b) initial reaction conditions are identified in the legend. Data is plotted as the % of bound peptide detected at a certain time point. Reactions were performed in triplicate, and data points represent one of two independent experiments. (c) DM-mediated dissociation of the CLIP peptide from DR1. The nature of the exchange peptide present in excess during the reaction is identified in the legend. Data points represent the mean of three independent experiments, and lines represent the fit of the data to a five parameter double exponential decay function. Due to the small SD, error bars are hidden below data points.

As shown in **Figure 3a**, we measured DR1/FAM labeled P7 L→C (HAC) complex stability under similar conditions as in the previous EPR assay. Again we observed a slow peptide off rate during the first 24 hours, which was greatly accelerated when exchange peptide, DM, or both were added to the respective reactions. Again, we observed a slight increase in off-rate during the first 24 hr in the presence of DM (18% loss) as compared to incubation in the presence of excess exchange peptide (3% loss), suggesting that the effect of DM incubation observed in the SDSL experiments was not an artifact of the experimental system used.

Given the importance of CLIP during the epitope selection process, we next asked whether the dependence of DM catalysis on the nature of the exchange peptide would be observed with CLIP prebound in the peptide binding groove. As shown in **Figure 3b**, we found similar results with DR1/CLIP complex stability in presence of DM.

We also investigated the relationship between exchange peptide affinity and release of prebound CLIP. For these experiments, DR1/CLIP complexes were used to test the effect of higher and lower affinity exchange peptides with respect to the peptide bound in the DR1 groove. As shown in **Figure 3c**, and consistent with the results presented in **Figure 1**, the rate of dissociation of the CLIP from DR1 varied considerably based on the affinity of the excess competitor peptide for DR1. When a high affinity exchange peptide such as wild type (wt) HA peptide was present in excess, the dissociation rate of the CLIP fitted to a double exponential decay, with a *t*_1/2_ of approximately 80 min. However, when the affinity of the exchange peptide was decreased either through P9 substitution (P9 L→P), P1 substitution (P1Y→A) or through multiple substitutions at positions with intermediate solvent accessibility (HASDPG), we found that the dissociation of the CLIP in the presence of DM was significantly reduced. None of the exchange peptides could promote a complete release of CLIP, since the largest CLIP/DR1 complex dissociation measured was on the order of 55% in the presence of excess HA. This observation may be related to the presence of a consistent fraction of complex unable to undergo the conformational changes needed for DM-mediated exchange. Interestingly, we found that as the affinity of the exchange peptide for DR1 decreased the relative contribution of the slow phase to the dissociation increases. During the slow phase, an equilibrium was established that appeared to be dependent on the affinity of the exchange peptide for DR1. For example, in the presence of an extremely low affinity exchange peptide such as HASDPG, the equilibrium is established at similar levels (≈90%) to that observed in the absence of exchange peptide.

### DM-mediated exchange is dependent on the concentration of the exchange peptide

**Figure 4:**
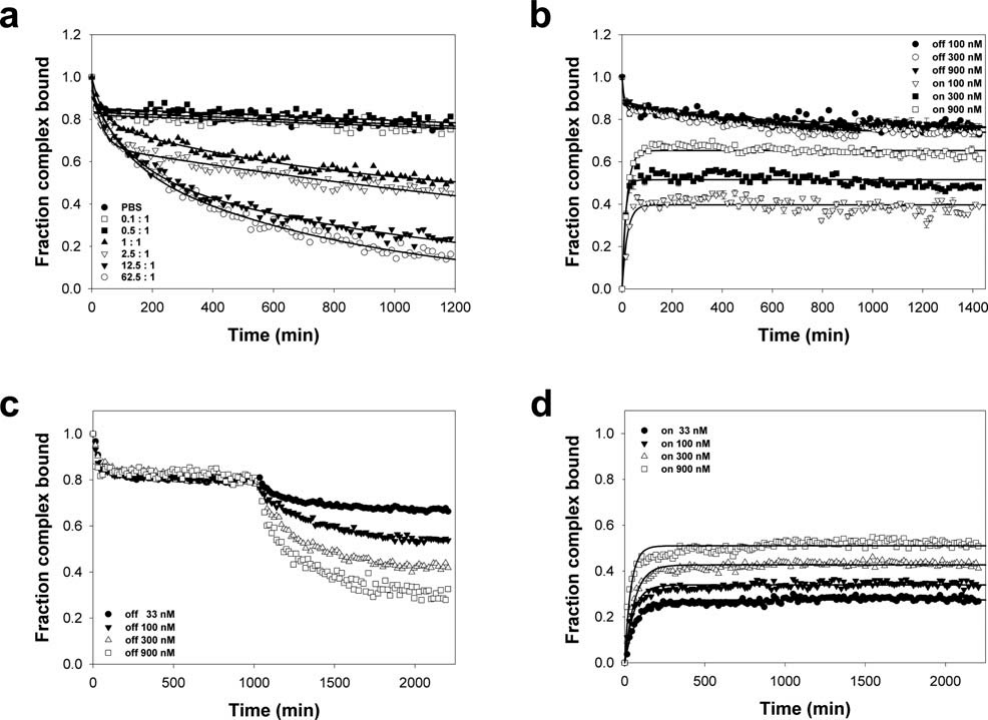
DM mediated peptide exchange as function of reactant concentration. (a) Requirement of equimolar exchange peptide for initiating exchange. DM-mediated dissociation of the peptide HAS from DR1 measured in the presence of different concentration of unlabeled HA in excess as described in Results. The exchange peptide to complex ratio for each reaction is identified in the legend. Data points represent the mean and SD of three independent experiments, and lines represent the fit of the data to a five parameter double exponential decay function. (b) FP analysis of DM-catalyzed peptide binding to and release from DR. CLIP/DR complex at different concentrations (100, 300, 900 nM) was incubated with 3 fold DM and allowed to dissociate in absence of any free peptide. Simultaneously, loading of FAM-CLIP to an equimolar amount of DR at the same concentrations was measured. Reactions were set up in triplicates, and the average ±SDs are shown. Lines represent the fit of the data to either a five parameter double exponential decay or four parameters double exponential raise function. (c) Peptide release in the absence of exchange peptide is not a function of complex concentration. FP analysis of DM-catalyzed release of HAD from DR at four different concentrations in absence of any free peptide. At *t* = 1000 after steady state was reached, unlabeled peptide was added at an equimolar concentration to the complex at *t* = 0. Reactions were set up in triplicates, and the average values for each time point are shown. (d) DM-mediated binding is a function of reactant concentration. FP analysis of DM-catalyzed association of HAD to equimolar empty DR at the same four different concentrations as in panel B. Lines represent the fit of the data to a four parameter double exponential function. For (c) and (d), due to the small SD, error bars are hidden below data points.

We observed that DM efficiently mediates the release of the prebound peptide during the exchange reaction only in the presence of an exchange peptide. Furthermore, the prebound peptide off-rate depends on the nature of the exchange peptide. By logical extension, if exchange requires a peptide of a certain affinity, then concentration of the reactants could be important. To address this question, we measured DM-catalyzed release of HAS from DR1 in the presence of different concentrations of unlabeled HA. As shown in **Figure 4a**, HAS dissociation varied considerably based on the concentration of the exchange peptide. While no significant release was measured for lower concentrations (0.1 and 0.5 fold relative to the peptide/MHCII complex), peptide exchange was observed at a 1∶1 ratio and higher.

An alternative explanation for the apparent stability of the peptide/MHCII complex in the absence of competitor peptide might be that the unlabeled peptide serves to prevent rebinding of the freshly dissociated pre-bound peptide. Under this model, the slower off rate could be due to formation of an equilibrium between dissociation and re-association. In our experiments, the initial complex concentration is very low, reducing the probability of re-assocation by simple Brownian motion in solution. However, we directly tested this possibility by measuring the stability of the CLIP/DR complex at three initial concentrations spanning one logarithm (100, 300, 900 nM), in the presence of DM without exchange peptide. Simultaneously, we measured the DM-mediated on rate of FAM-CLIP to an equimolar amount of DR at the same concentrations. We reasoned that as the concentration of both DR and peptide increased, the probability of peptide binding to DR should increase accordingly as measured by the plateau of the peptide on-rate. A corollary is that for low concentration samples, the on-rate steady state is a fraction of that detected in the corresponding release assay.

As shown in **Figure 4b**, DM-mediated CLIP release in the absence of any unlabeled peptide is essentially the same for all the concentrations tested, reaching an equilibrium at 80% of bound peptide. This indicates that the fraction of bound (and free) peptide at any time-point is not a function of the initial complex concentration. On the contrary, during a loading assay, the fraction of bound peptide at the steady state depends on peptide and DR concentration, as predicted. In particular, at 100 nM, which corresponds to the concentration usually adopted in our experiments, a significant difference between peptide release and peptide binding at equilibrium is evident; thus, the plateau established during a peptide off rate can not be explained simply as a cycle of release and rebinding. Moreover, this provides further support for the model that the unlabeled peptide is not only replacing the pre-bound but actively participating in DM action. Interestingly, DM contribution to peptide association appears more significant than DM contribution to peptide release, as the effect of DM on complex formation is greater than complex dissociation for all the samples tested.

On the basis of this result and the finding that free peptide concentration affects complex stability (**Figure 4a**), we speculated that the kinetics during peptide release in the presence of DM is determined by the rate at which DM promotes the loading of the free peptide. To test this hypothesis we compared the DM mediated release of the prebound peptide in the presence of a specific concentration of exchange peptide with the DM mediated association of the exchange peptide to empty DR at that same concentration.

As shown in **Figure 4c**, four different concentrations of DR1/HAD complex were allowed to reach equilibrium in the presence of 3 fold excess DM and in the absence of exchange peptide. Under these conditions, the small fraction of HAD released from DR1 is essentially the same for all the concentrations of DR1/HAD complex tested. As observed for CLIP, the percentage of bound (and free) peptide at any particular time-point in the absence of exchange peptide is not a function of the initial complex concentration. Next, unlabeled HAD peptide was added in equimolar amounts with respect to the initial complex concentration. For the results reported in **Figure 4a**, a 1∶1 exchange peptide as to complex ratio is sufficient to promote peptide exchange. As shown in **Figure 4c**, the rate of peptide exchange was dependent on the absolute concentration of exchange peptide, despite being present at equimolar amounts. Interestingly, we found that the extent of DR1/peptide complex depletion was related to the maximal plateau of DR1/peptide complex formation in the presence of DM (**Figure 4d**), indicating that the ability of DM to mediate peptide exchange is dependent on the ability of exchange peptide to bind DR1 in the presence of DM.

### Direct analysis of cooperativity in DM-mediated peptide association

**Figure 5:**
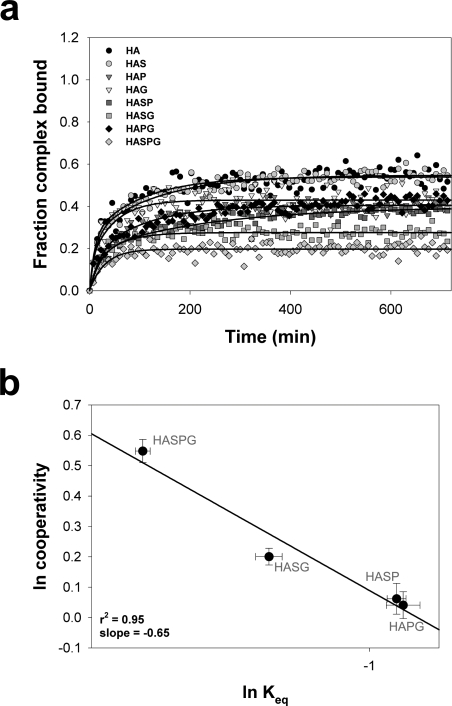
Direct measure of cooperativity in DM-mediated peptide association. (a) Association rate of peptides to DR1 in the presence of DM was measured as described in Results. Data is plotted as the fraction of either peptide or DR1 forming complex. Reactions were performed in triplicate, and data points represent one of three independent experiments. Error bars are omitted for graphic clarity. Lines fit the data to a four parameter double exponential function. (b) Natural log (ln) plot of cooperativity (expected/observed *K*_eq_) vs. association rate for each peptide tested. Error bars are as in figure 1b. The line indicates the fit of the data to a linear regression.

One possible explanation for these data is a mechanism by which DM is sensitive to the presence and nature of possible epitopes, thereby actively facilitating of the binding of the exchange peptide. Indeed, the cooperativity measured at the level of the exchange peptide as shown in **Figure 1(c–f)** is indirect evidence for DM mediated peptide/MCHII folding. To directly measure cooperativity in DM-mediated binding of peptide to DR1, we utilized FP to monitor the loading of different peptides to an equimolar amount of empty MHCII (100 nM) in the presence of a 3 fold excess of DM (**Figure 5a**). In these experiments, cooperativity was calculated as expected to observed ratio of the normalized *K*_eq_. As shown in **Figure 5b**, plotting cooperativity against the observed *K*_eq_ of each peptide, an exponential relationship was revealed, with cooperativity increasing as peptide association decreased (slope −0.65). Thus, the cooperativity we observed in the release of prebound peptide as the affinity of the exchange peptide was varied was replicated when we directly measured the DM-mediated binding of the exchange peptide into empty DR.

### Co-localization of prebound and free peptide during DM-mediated exchange

Given the dependence of DM-mediated peptide exchange on the presence of an exchange peptide, the mechanism of DM-mediated peptide exchange requires three molecular species; the prebound peptide/MHCII complex, exchange peptide, and DM. One possible mechanistic explanation for our findings would be the formation of a transient two-peptide/MHCII intermediate during the exchanging reaction. Indeed, evidence for formation of a two-peptide/MHCII intermediate in the absence of DM has previously been reported [19], [30]. Measurement of fluorescence resonance energy transfer (FRET) from aromatic residues in the MHCII protein to labeled peptide side chains has been previously utilized to monitor peptide binding, complex dissociation, and the formation of a two-peptide/MHCII complex [19], [30], [31]. In general, the intermolecular distance between fluorescent donor and acceptor determines the strength of the FRET signal. Therefore, we asked whether we would observe a measurable FRET signal between the exchange and prebound peptide during the DM-mediated exchange reaction.

**Figure 6:**
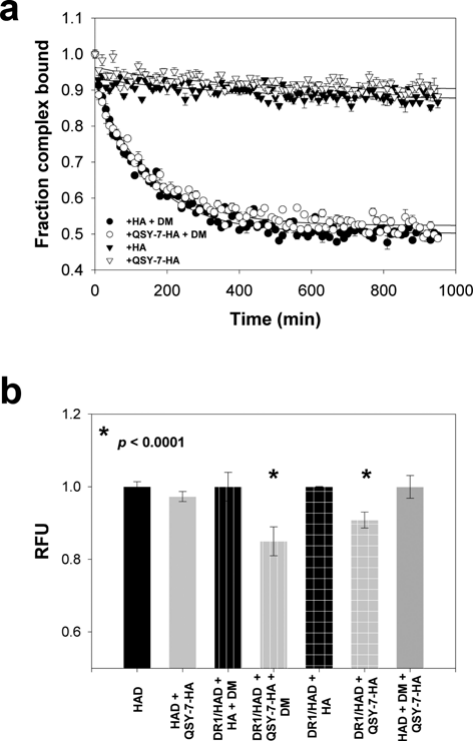
Co-localization of prebound and free peptide during DM-mediated exchange. (a) DM-mediated and intrinsic dissociation of HAD from DR1 was measured as described in Methods. The nature of the exchange peptide present in excess during the reaction is identified in the legend. Data points represent the mean of three independent experiments, and lines represent the fit of the data to a five parameter double exponential decay function. (b) Reduction of the overall fluorescence due to FRET between FAM-labeled HAD and QSY7-HA (shaded bars) either with (striped bars) or without (squared bars) DM as compared with the fluorescence detected in presence of unlabeled free HA peptide during the dissociation of HAD from DR1 (dark bars). Plain bars represent the fluorescence detected in the following reactions: HAD (dark bar), HAD+QSY7-HA (shaded bar) and HAD+QSY7-HA+DM.

To address this question, we formed DR1 complexes with N-terminally FAM-labeled HA peptides containing the P3 (K→D) mutation. Next, we constructed an exchange peptide consisting of the wild type HA sequence labeled with a quencher probe (QSY-7-HA) at the N-terminus. QSY-7 is a non-fluorescent diarylrhodamine derivative that has strong absorption in the FAM emission spectrum without direct acceptor excitation. The presence of the QSY-7 probe had minimal (3-fold) effects of HA affinity for DR1 as shown by a competitive binding assay (data not shown). We then measured the off rate of HAD in the presence of QSY-7-HA and unlabeled wild-type HA in the presence and absence of DM. As shown in **Figure 6a**, the presence of the QSY-7 moiety did not significantly affect the ability of the wild-type HA peptide to compete for the DR1 binding groove in the presence or absence of DM.

We next measured the ability of the QSY-7-HA to quench the FAM fluorescence in the presence or absence of DM. As shown in **Figure 6b**, we detected a 20% decrease in fluorescence signal during the HAD off-rate performed in the presence of QSY-7-HA and DM as compared to the control (p<0.0001), indicating that a significant energy transfer was occurring during the exchanging reaction. Interestingly, the fluorescence decreased by 10% as compared to the control when performed in absence of DM, indicating a less frequent co-localization during intrinsic peptide release (p<0.0001). This difference was not due to either random collisions between free peptides or a nonspecific action of DM on the two peptides, as no significant loss of fluorescence was measured in either of these cases. These data suggest that during peptide release, there is a time when the two peptides are within 35–55 Å of each other, and suggest that transient formation of a two peptide/MHC intermediate is enhanced in the presence of DM.

## Discussion

Despite its importance in epitope selection, the mechanism of DM-mediated peptide exchange remains unclear. In this work, we show that the efficient release of the prebound peptide during a DM-mediated exchange reaction requires the presence of an exchange peptide. Furthermore, the ability of the exchange peptide to act as a co-factor in the displacement is dependent on its ability to bind MHCII, as judged by affinity. Moreover, the exchange reaction requires a threshold concentration of the exchange peptide. Enhanced cooperative effects measured during peptide dissociation in the presence of DM are observed at the level of the exchange peptide, not at the level of the prebound peptide. Cooperativity is also observed at the level of DM-mediated peptide binding in the absence of a prebound peptide. Finally, in the presence of DM, there is an increased frequency of co-localization of the exchange and prebound peptides interacting with MHCII molecules.

Taken together, our data support a “compare-exchange” [39] mechanism of DM action in which the presence of equimolar or greater exchange peptide promotes a short lived tetramolecular interaction involving DM, the MHCII-prebound peptide complex and the exchange peptide. DM changes the structure of the MHCII molecule, resulting in an extremely rapid release of the prebound peptide. This release is not based on a gradual unfolding process. It would include a large scale disruption of the hydrogen bond network at the N-terminus of the peptide/MHCII binding groove [16]–[18] and a weakening of the hydrophobic interaction at P1, leading to wide-scale disruption of peptide/MHCII interactions throughout the binding groove. Although destabilized, the prebound peptide remains in the complex while DM maintains the MHCII in an energetic state sensitive to the folding properties of the exchange peptide. In the absence of productive folding of the exchange peptide, the original prebound peptide can rebind to the groove. The end result is that DM selects for exchange peptides with the best chance of binding based on their affinity.

A key aspect of this mechanism is presence of a metastable transition state in which two peptides may be accommodated in close proximity to the binding groove. Some evidence in support of a two-peptide/MHCII transition state is provided by FRET experiments in which peptide to peptide energy transfer was detected only in the samples containing a preformed complex and an exchange peptide. Further support for this model is provided by kinetic stability of peptide/MHCII complexes either in the presence of DM or the absence of an exchange peptide. Previously reported data in which a second peptide helps to release (or load) another peptide based on its affinity [19], [30], [33] also support a transient two peptide/MHCII state. Although estimates of the relative proportion of the two-peptide/MHCII complex were low in those studies, (1.0–0.1%), these complexes were preferentially associated with the “open” conformer of the peptide/MHCII complex during native PAGE analysis [30]. In keeping with the reported correlation of two peptide intermediates and “open” conformers, we propose that the DM-associated two-fold increase in interpeptide FRET indicates that DM senses the “open” MHCII resulting from the interaction with the two peptides.

If cooperative effects in the peptide association and dissociation to MHCII in the absence of DM are directly related to coordinate folding of the peptide and MHCII [21], [22], then the lack of cooperativity in DM-mediated peptide dissociation is striking, and suggests that DM promotes a dramatic structural change in the peptide/MHCII complex that does not follow the usual energetic pathway of peptide/MHCII folding. One possibility is that DM may promote a transient but catastrophic destabilization of the pre-bound peptide/MHCII complex, possibly through alteration of the three hydrogen bonds mediated by residues (51–53) of the MHCII α chain [16] and 81 of the β chain [17], [18]. This structural change at the P1 region may then be transmitted rapidly throughout the entire length of the peptide binding groove such that the typical peptide/MHCII unfolding is absent, as evidenced by the lack of measurable cooperativity. Under these conditions of widespread disruption of peptide/MHCII interactions, the probability of close approximation of the prebound and exchange peptides to a destabilized peptide binding groove is enhanced.

The subsequent peptide comparison and binding step of the exchange reaction can be considered as either a stochastic competition for the binding groove of the MHCII, or an ordered process with the geometry of the retained pre-bound peptide and the position of the exchange peptide favoring the latter's access to the groove. In either scenario, DM would promote the folding of the peptide/MHCII complex to the final conformation. In a stochastic competition, both peptides would simultaneously attempt to fit into the groove, and half of these events would result in rebinding of the original peptide when the two peptides show the same affinity. In an ordered model, the exchange peptide would be tested first and only if it was incapable of binding would the prebound peptide return to the groove. Although we are pursuing experiments to discriminate between these possibilities, we do observe a difference in the cooperativity of binding in the presence of the prebound peptide (**Figure 1d**, slope −0.99) vs. that observed in the absence of the prebound peptide (**Figure 5b**, slope −0.65) using largely empty soluble DR1 molecules. One explanation for this difference would be that the by forming an initial complex, the prebound peptide shifts the peptide/MHCII complex into a conformer receptive to subsequent efficient cooperative folding in the presence of a competitor peptide. *In vivo*, the CLIP peptide may play a similar role.

We should point out that the selection of which peptide will fold into the MHCII is restricted to the two peptides in the complex. The MHCII is unavailable to third party peptides, as the experiments shown in **Figures 1a–b**, as well as **Figure 3c** are performed adding a large excess of exchange peptide and clearly no mass action effect can be detected for peptides with intrinsic low affinity for MHCII.

Mapping the location of the exchange peptide on the peptide/MHCII complex in the presence or absence of DM will be an important step in refining the mechanism. Due to the need for diverse competitor peptide recognition, the most likely possibility is that the incoming competitor peptide may associate with the exchanging complex by forming partial hydrogen bond or hydrophobic interactions with the destabilized peptide binding groove. As the amino acid polymorphism in the peptide binding groove across different MHCII alleles result in “anchor-pocket” interactions of varying strength, we expect that hydrogen bonding may provide the majority of the binding energy for competitor peptide recognition. However, we cannot entirely exclude the possibility that the competitor peptide interacts with a distinct (presumably less polymorphic) site present across MHCII alleles. Experiments to chemically cross-link the competitor peptide during the exchange reaction may provide some information regarding the structure of the exchanging complex. An alternative approach may be to examine mutagenized MHCII molecules for their ability to undergo peptide exchangeability in the absence or presence of DM [13].

Interestingly, we found that DM could promote a small, yet measurable peptide release in absence of an exchange peptide. Furthermore, this activity was independent of concentration (**Figure 4b**). The phenomenon is likely related to the presence of multiple conformers of the peptide/MHCII complex. At least two isomers have been hypothesized, of which one would be responsible for the slow phase and one for the fast phase of the peptide release reaction [35]–[37]. Moreover, it has been proposed that DM might distinguish between these isomers [32]. One possibility is that in the presence of DM and absence of an exchanging peptide we observe peptide dissociation from the “fast release” conformers, on which the weak destabilizing action of DM would be enough to promote peptide release. The “slow release” isomers require an exchanging peptide for peptide exchange. Experiments are currently underway to test this hypothesis.

One limitation of the current study is that a single MHCII allele was used in the experiments. Therefore, further experiments must be conducted to confirm a common mechanism of DM-mediated peptide exchange across various MHCII alleles. If DM acts to promote peptide binding groove destabilization through disruption of peptide/MHCII interactions near the P1 pocket, the effect of MHCII P1 polymorphism may also provide additional insights into the mechanism of DM-mediated exchange. Preliminary experiments with other human MHCII alleles confirm the presence of cooperativity in the absence of DM, supporting the hypothesis that the total distributed binding energy available to the peptide/MHCII complex contributes to complex formation, whether from hydrogen bonds or hydrophobic “anchors”. Therefore we do not anticipate the need of an alternative mechanism to explain the outcome of DM interaction with different MHCII alleles.

How might the “compare-exchange” mechanism be applied to our current understanding of epitope selection *in vivo*? Based on our data, an attractive hypothesis would be that DM evolved to accelerate the process of generating the highest stability peptide/MHCII complexes within a given pool of available peptide sequences within the MIIC. Currently, it is unclear how many cycles of peptide exchange a peptide/MHCII complex undergoes prior to egress from the MIIC. A specialized cellular substructure has been identified in which such decisions may be made [38], but on what basis is still unclear. One can envisage termination of the exchange reaction based on generation of a true DM-mediated “compact” conformation. The answer may lie in elucidating the molecular details of the resolution of the tetramolecular complex. The “compare-exchange” mechanism proposed here for DM mechanism might also be important for antigen processing via the MHC class I pathway. MHC class I processing involves proteins structurally related to MHCII, and both classes of MHC molecules undergo peptide-dependent conformational change. Furthermore, the majority of MHC class I alleles requires the intervention of the tapasin-ERp57 heterodimer to optimize their peptide cargo [40]. The “compare-exchange” mechanism may provide additional insights into the biology of MHC antigen processing, and may also be generally applicable to other biological systems in which protein receptors must bind diverse yet structurally related ligands.

## Materials and Methods

### Peptide Synthesis

Peptides derived from the sequence GPKYVKQNTLKLAT, representing residues 306–318 of the hemagglutinin protein from influenza A virus (H3 subtype), are described in table 1. The N-terminal Gly facilitated labeling. Side chains in the HA peptide are numbered relative to the P1 Tyr residue [41]. The sequence PVSKMRMATPLLMQA represents residues 87–101 of the CLIP peptide [42]. Peptides were synthesized by standard solid-phase methods, purified by HPLC, and confirmed by mass spectrometry. N-terminal labeling with FAM (Molecular Probes) or LC-LC biotin (Pierce) was performed on the resin before deprotection, and then peptides were cleaved and purified by HPLC. For the spin labeling, the HA peptide sequence was substituted with Cys at P1, P3 or P7, synthesized using FMOC chemistry and standard protocols, purified by HPLC and confirmed by MALDI-TOF mass spec (Protein Nucleic Acid Shared Facility- MCW). Subsequently, 10 mg of the sulfhydryl-specific EPR probe MTSL ((1-oxyl-2,2,5,5-tetramethyl-3-pyrroline-3-methyl) methanethiosulfonate spin label; Toronto Research Chemicals) previously dissolved in 200 μl of DMSO was added to 20 mg of purified peptide dissolved in 10 ml of 5% acetic acid. After 5 h of incubation (dark, rocking, room temperature), the coupling was monitored by RP-HPLC, the peptide re-purified by RP-HPLC and verified by MALDI-TOF mass spec. N-terminal labeling with QSY-7 (Molecular Probes) of wtHA was performed according to the manufacture's protocol after peptide purification. Unbound probe was purified by HPLC.

### Expression and Purification of Recombinant Soluble DR1 and DM Protein

Recombinant soluble empty (peptide free) DR1 was produced and immunoaffinity purified from a stably transfected *Drosophila* S2 insect cell line essentially as described [43]. It is known that DR1 produced in S2 cells might have insect-derived peptide loosely bound once purified. Nevertheless the increased peptide binding capacity, increased binding rate and decreased pH dependence of peptide binding as compared to mammalian-derived DR1 indicate that, as isolated, the antigen binding site is largely empty (>85%) [43]. Soluble FLAG epitope tagged DM was isolated from a stably transfected *Drosophila* S2 cell line as described [9]. To avoid contamination with FLAG peptide, DM elution from the resin was performed with 0.1 M glycine HCl, pH 3.5. Both DR1 and DM proteins were purified and buffer exchanged into PBS (7 mM Na^+^/K^+^ phosphate, 135 mM NaCl, pH 7.4) using centrifugal ultra-filtration (Amicon). Purity (>95%) was confirmed by SDS-PAGE stained with GelCode Blue Stain Reagent (Pierce). DR1 and DM proteins were quantified by measuring the UV absorbance @ 280 nm using an E_280_ of 56340 M^−1^ cm^−1^ before use.

### PAGE analysis of DM Mediated and Intrinsic Peptide Dissociation

DR1/peptide complexes were formed by incubating 1 μM DR1 protein with a 10-fold molar excess of FITC-labeled peptide in 50 mM NaH_2_PO_4_ and 50 mM of sodium citrate (pH 5.3) and protease inhibitors for 16 h @ 37°C. DR1/peptide complexes were then purified from unbound peptide by buffer exchange into PBS with a Centricon-30 spin filter that had been pre-incubated with 25 mM MES (pH 6.5). Purified DR1/peptide complexes were then quantified by reading the UV absorbance @ 280 nm, factoring in an *E*_280_ of 1280 M^−1^ cm^−1^ for the Tyr residue and 10846 M^−1^ cm^−1^ for the fluorescein present in the bound peptide. Purified DR1/peptide complexes (85 nM) were then incubated with 10 μM unlabeled HA peptide @ 37°C in 50 mM NaH_2_PO_4_ and 50 mM of sodium citrate (pH 5.3).To prevent nonspecific adherence of the protein, siliconized tubes were used. At various time points, aliquots of the reaction were removed and quenched with 0.5 M Tris-HCl (pH 8.0) in gel loading buffer and immediately placed on ice. The aliquots were then loaded onto a 5/12% native PAGE gel and quickly separated by electrophoresis at 150 V for 30 min. FITC-peptide/DR1 complexes were then visualized using a FluorImager (Molecular Dynamics). Data was normalized and expressed as the % of FITC-peptide/DR1 complex remaining relative to the complex at *t* = 0, and fit to a single or double exponential model. Each experiment was performed in triplicate, and the reported dissociation rate reflects the mean±SD of three independent experiments.

### Fluorescence Polarization Dissociation and Association Measurements

DR/peptide complexes were formed by incubating 1 μM DR1 with a 10-fold excess of FAM-labeled peptide as described above, and purified from unbound peptide by buffer exchange into PBS (pH 7.4) with a Centricon YM-30 spin filter (Amicon). 100 nM of purified DR1/peptide complexes were incubated with 100-fold excess of unlabeled HA 306-318 peptide in the presence of 3-fold excess DM. In some experiments either the sequence or the concentration of the exchange peptide was varied. Reactions were performed @ 37°C in 50 mM sodium citrate/sodium phosphate buffer at pH 5.0–5.3 and were covered with mineral oil to prevent evaporation. For the association assay, equimolar DR1 and FAM-labeled peptide were co-incubated with 3-fold excess of DM. Fluorescence polarization was monitored after addition of the peptide and DM until equilibrium was reached. To avoid non-specific adherence of the protein, black polystyrene 96-well plates were used (Corning). Measurements were performed using a Wallac VICTOR counter (PerkinElmer Wallac) with the excitation wavelength = 485 nm and emission wavelength = 535 nm. Specific control groups included (a) protein only, (b) peptide only, and (c) buffer only, and were used for background correction. FP and anisotropy are mathematically related ways of expressing parallel:perpendicular emission ratios and are easily interconverted. Although FP is approximately linear with respect to the ratio of free∶bound peptide, FP was converted to anisotropy (which is exactly linear) by the following equation A = 2*FP/(3−FP) where A is anisotropy and FP indicates fluorescence polarization in mP units. Anisotropy values were fitted either according to a single- or a bi- exponential decay model. Each experiment was performed in triplicate, and the reported dissociation rate reflects the mean±SD of three independent experiments.

### Competitive Peptide Binding Assay

DR1 (20 nm) was incubated with 20 nm biotinylated HA peptide in PBS (0.1% BSA, 0.01% Tween 20, 0.1 mg/ml 4-(2-aminoethyl)-benzene sulfonyl fluoride, 0.1 mM iodoacetamide, 5 mM EDTA, 0.02% NaN_3_, pH 7.2) in the presence of varying amounts of inhibitor peptides for 3 days at 37°C. The incubation time ensures the majority (>65%) of DR1 protein participates in the peptide binding reaction to reach equilibrium. Bound biotinylated peptide was detected using a solid-phase immunoassay and Eu^2+^ labeled streptavidin [44]. Plates were read using a Wallac VICTOR counter (PerkinElmer Wallac). Data was fit to a logistic equation *y* = *a*/[1+(*x*/*x*_0_)*^b^*]. IC_50_ values were obtained from the curve fit of the binding data and converted to *K*_D_ values by using the equation *K*_D_ = (IC_50_)/(1+[bHA]/*K*_D,bHA_)) in which *K*_D,bHA_ was set equal to 14 nM on the basis of the results of the direct binding of bio-HA peptide to DR1. Each point represents the mean and SD of three independent experiments performed in quadruplicate. Because peptide/MHCII binding represents a multistep reaction, the IC_50_ for a competitive binding assay may not be directly proportional to the *K*_D_. While this can be offset by long incubations relative to half life, we study low affinity peptides where half-lives are impossible to determine. Therefore, the values of affinity reported herein should be considered as apparent *K*_D_ values.

### EPR Measurements

The spin labeled peptide was bound to DR1 for 3 days @ 37°C in acid buffer (pH 5.0) and the unbound peptide removed by twelve cycles of ultrafiltration through a Centricon YM-30 spin filter (Amicon) previously incubated with 200 μl of 25 μM MES. 25–30 μM of purified DR1/peptide complex were incubated for 24 hr with either 100-fold molar excess of unlabeled HA 306-318 peptide or 3-fold molar excess of DM or in their absence as a control. After 24 hr, 3-fold excess of DM or 100-fold excess of unlabeled exchange was added to the first two reactions. X-band EPR spectra of the three samples (∼20 μL) contained in glass capillaries were recorded on a Bruker ElexSys E500 fitted with a super high Q cavity. Spectra were acquired at room temperature over a 100 G range with an incident microwave power of 10 mW at the following timepoints: 0, 24 24.5, 25.5, 26.5, 27.5, 28 hr. After 47 hr DM and unlabeled HA were added to the control and spectra of this sample were acquired as above. Each timepoint typically represents the average of 9–25 scans collected over 6–18 min, respectively, and is plotted according to the time at which the first of the averaged scans was recorded. Spectral analysis to obtain the percentage of free peptide for each timepoint was carried out using spectral subtraction and double integration methods with software kindly provided by Dr. Christian Altenbach (University of California, Los Angeles).

### Calculation of Cooperative Effects

We utilized a multiple substitution strategy previously used to identify interacting partners during protein folding [23], [26]. To normalize the *t*_1/2_ values of a given peptide/MHCII complex, we define the effect of each substitution as the ratio of the substituted measurement over that of the DR1/wtHA value (Δ*t*_1/2_). For calculating cooperativity, the effect of multiple substitutions is measured directly (observed value). The expected value for a combination of substitutions is calculated as the product of the individual substitutions [e.g. Δ*t*_1/2,exp_ *x,y* = (Δ*t*_1/2_, *x*)×(Δ*t*_1/2_, *y*)]. For peptides with three substitutions, the expected value would be the product of all the different substitutions [e.g. Δ*t*_1/2,exp_ *x,y,z* = (Δ*t*_1/2_, *x*)×(Δ*t*_1/2_, *y*)×(Δ*t*_1/2_, *z*)]. The cooperativity is the ratio of the expected to observed (*C* = exp/obs) values for Δ*t*_1/2_. A value of 1 for the ratio of expected/observed indicates no cooperativity, for it would suggest independent energetic contribution from each substitution. Cooperativity is evidenced when the ratio of expected/observed is not equal to 1. Since each measurement (both expected and observed) is affected by an error and cooperativity is calculated as their ratio, its value is affected by the propagation of the relative errors.

Thus, error on cooperativity is calculated through standard error propagation: 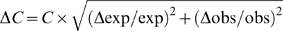 and, in a ln plot, the error is calculated as: Δ*C*_ln_ = Δ*C*/*C*.

### FRET

The extent of a possible bi-peptide colocalization was measured fluorometrically using a 5-carboxyfluorescein (5-FAM)/QSY-7 fluorescence resonance energy transfer (FRET). QSY-7 is a non-fluorescent diarylrhodamine derivative that has strong absorption in the FAM emission spectrum without direct acceptor excitation. QSY-7 extinction coefficients are typically in excess of 90,000 cm^−1^M^−1^and absorption spectra of the conjugates are insensitive to pH between 4 and 10. Fluorescence quantum yields are typically <0.001 in aqueous solution. QSY-7 presents high chemical stability of the conjugates and very good resistance to photobleaching. In a standard FRET-based proximity study, the residual fluorescence of 5-FAM is recovered and can be monitored at excitation/emission wavelengths of 480±10/520±10 nm. DR1/peptide complexes were formed by incubating 1 μM DR1 with a 10-fold excess of FAM-labeled peptide and purified as described above. 100 nM of purified DR1/peptide complexes were incubated with 100-fold excess of QSY-7 labeled HA 306-318 peptide in the presence of a 3-fold molar excess of DM. Specific control groups included (a) FAM-HA only, (b) FAM-HA and QSY-7-HA, (c) FAM-HA, QSY-7-HA and DM, (d) buffer only (e) protein only, with (d) and (e) used for background correction. The change in fluorescence (expressed as relative fluorescence units) was monitored at 5-min intervals for 1 h @ 37°C using a Wallac VICTOR counter (PerkinElmer Wallac) with an excitation wavelength = 485 nm and an emission wavelength = 535 nm. Data were analyzed using an unpaired Student's *t* test (two groups). In all statistical tests, differences were considered significant when *p*<0.05. Data are presented as mean±S.E.M. Statistical analysis was performed using the program SigmaPlot for Windows, version 9.0 (Systat Software, Inc., San Jose, CA).
